# Long-term satellite tracking reveals patterns of long-distance dispersal in juvenile and adult Arctic foxes (*Vulpes lagopus*)

**DOI:** 10.1098/rsos.220729

**Published:** 2023-02-01

**Authors:** Richard Gravel, Sandra Lai, Dominique Berteaux

**Affiliations:** Canada Research Chair on Northen Biodiversity, Centre for Northern Studies and Quebec Centre for Biodiversity Science, Université du Québec à Rimouski, Rimouski, Canada G5L 3A1

**Keywords:** tundra, breeding dispersal, canid, movement ecology, natal dispersal, sea ice

## Abstract

Long-distance dispersal plays a key role in species distribution and persistence. However, its movement metrics and ecological implications may differ whether it is undertaken by juveniles (natal dispersal) or adults (breeding dispersal). We investigated the influence of life stage on long-distance dispersal in the Arctic fox, an important tundra predator. We fitted 170 individuals with satellite collars during a 13-year study on Bylot Island (Nunavut, Canada), and analysed the tracks of 10 juveniles and 27 adults engaging in long-distance dispersal across the Canadian High Arctic. This behaviour was much more common than expected, especially in juveniles (62.5%, adults: 19.4%). Emigration of juveniles occurred mainly at the end of summer while departure of adults was not synchronized. Juveniles travelled for longer periods and over longer cumulative distances than adults, but spent similar proportions of their time travelling on sea ice versus land. Successful immigration occurred mostly in late spring and was similar for juveniles and adults (30% versus 37%). Our results reveal how life stage influences key aspects of long-distance dispersal in a highly mobile canid. This new knowledge is critical to understand the circumpolar genetic structure of the species, and how Arctic foxes can spread zoonoses across vast geographical areas.

## 1. Introduction

Dispersal is a major process in ecology and evolution, playing a key role in species distribution and persistence [1]. Defined as the movement of an individual from its natal to its first breeding area (natal dispersal) or between successive breeding areas (breeding dispersal) [2], dispersal is largely involved in population dynamics, gene flow and the transmission of diseases and parasites [3–5]. One type of dispersal, long-distance dispersal, is of particular interest given its potential to profoundly reshape the distribution and genetic variability of species. Long ago, Darwin [6] devoted two chapters of The Origin of Species to discuss long-distance dispersal and its implications for species distribution. This form of movement was defined alternatively as dispersal beyond a certain threshold distance (based on ecological knowledge of the species), or over distances that are substantially longer than those reached by most individuals in the population [7].

Although long-distance dispersal has the potential to play a disproportionate role in population demography and genetics, its rarity and unpredictability make it very difficult to quantify in most species [7–9]. The study of long-distance dispersal has therefore long been associated with high complexity and uncertainty [7]. In vertebrates for example, despite the astonishing progress of radiotracking technologies [10], the high cost of tracking many individuals [11] combined with the often high mortality rate of dispersing juveniles [12] and the unpredictability of adult dispersal [13] make long-distance dispersal studies very difficult to conduct.

Dispersal involves three distinct stages, namely emigration (departure from the initial home range), transience (displacement between the initial and final home ranges) and immigration (settlement on the final home range) [14,15]. Tracking the whole dispersal process is crucial to understand dispersal mechanisms. Indeed, individuals undertaking dispersal go through different behavioural states, influencing movement metrics such as the timing, daily rate, length and linearity of movements [16–18]. Life stage may also have an important influence on movement patterns during dispersal [18–20]. Because of their lack of experience and a higher propensity of being subordinate, juveniles are likely to have more difficulties than adults to settle in a high-quality patch following long-distance dispersal [14,21]. Consequently, a general trend is that juveniles travel greater distances during natal dispersal than adults do during breeding dispersal [2,18,22–25]. Timing of emigration may as well change with life stage because of differential drivers of dispersal. For juveniles, inbreeding avoidance and competition for space may synchronize emigration dates [26]. Specifically, emigration events should peak when individuals become independent and undergo increased sibling or parent-offspring agonistic behaviours [27–30]. By contrast, adults may emigrate when the sum of the reproductive value of their offspring surpasses their own reproductive value [31] or when they lose their territory to a competitor, which might happen at different times of the year. Regarding immigration, both juveniles and adults should settle before the start of the breeding season to minimize the costs of skipping a reproduction event or reproducing with a lack of site experience [32,33].

Most studies investigating the influence of life stage on long-distance dispersal have been conducted on birds and fewer of them concern mammals. Dispersal is nonetheless omnipresent in terrestrial mammals and long-distance dispersal is notably observed in carnivores, which usually travel greater distances than herbivores and omnivores [34]. Canids are especially highly mobile, and records of the longest annual distances travelled by terrestrial mammals are often found within this family group [35]. Canids also show high variability in dispersal patterns, likely due to differences between species in body size, diet and resource availability [36]. Notably, despite their small size, Arctic foxes (*Vulpes lagopus*) show impressive long-distance dispersal, with trips of thousands of kilometres on land and ice being reported from tag recoveries [37] and satellite telemetry [38–41]. The 3 kg Arctic fox is a key terrestrial predator and facultative scavenger of the tundra showing extensive movements on sea ice [42]. Genetic studies suggest that circumpolar populations linked by sea ice are one panmictic population [43–45], testifying to the omnipresence of long-distance dispersal in this species.

Besides being a prime model for the study of long-distance dispersal, a better understanding of Arctic fox movements also has practical benefits because the species is a critical vector of the rabies virus and the *Echinococcus* tapeworm, both generating zoonoses and thus important public health concerns [46–49]. In addition, climate change is quickly reorganizing the phenology, distribution and structural characteristics of Arctic sea ice, with effects on Arctic biodiversity needing urgent attention [50]. Concerns have been raised regarding the population connectivity and movements of species, such as the Arctic fox, that use sea ice as a travelling platform [43,51]. Understanding the drivers of long-distance dispersal requires the study of both external (food, competition, habitat, etc.) and internal factors (sex, age, size, etc.). In particular, the respective contributions of juveniles and adults to long-distance dispersal represent a key knowledge need since dispersal in general is characterized by much greater participation of juveniles than adults. Yet, information about movements of juvenile Arctic foxes is largely lacking [40].

We used data from an extensive 13-year satellite tracking study involving 170 Arctic foxes in the Canadian High Arctic, to characterize long-distance dispersal in this species and test predictions about the effects of life stage on its three stages. Starting with the occurrence and immigration success of long-distance dispersal events, we predicted (P1a) that the proportion of individuals engaging in this type of movement should be higher in juveniles than in adults [26], but that juveniles should be less successful than adults at securing a new territory (P1b). Following up with the timing of long-distance dispersal, we predicted that juveniles should emigrate at the end of summer and thus be rather synchronized (P2a), whereas adults should emigrate at any time and thus without synchrony (P2b). We also predicted that successful immigrants should settle before the start of the breeding period, when pairs need to secure a territory where they will raise their young, that is no later than April (P2c) [52]. Then, regarding the transience stage, we predicted that juveniles should travel for longer periods of time (P3a) and over greater distances (P3b) than adults, but we did not expect differences between age groups regarding the linearity (P3c) or the daily rate (P3d) of movements [53]. When sample sizes allowed, we tested the effects of sex on the above-studied parameters, predicting (P4) no sex effects in this mainly monomorphic and monogamous species [2,52,54,55]. Finally, we investigated the direction of long-distance dispersal events in juveniles and adults, as well as their respective use of sea ice and land. With no basis to build robust predictions, these last objectives are mostly descriptive and aimed at generating hypotheses for further testing. Predictions are summarized in table 1.

**Table 1.** Predictions related to the long-distance dispersal of juvenile and adult Arctic foxes from Bylot Island (Nunavut, Canada). Characters indicate whether predictions were supported (bold), partly supported (italics) or not supported (regular) by our results.

		predictions	Ref
P1	**a**	**occurrence of emigration leading to long-distance dispersal is higher in juveniles than in adults**	[26]
	b	occurrence of successful immigration following long-distance dispersal is lower in juveniles than in adults	[14,21]
P2	**a**	**juveniles should emigrate at the end of summer and thus be rather synchronized**	[26–30]
	b	*adults should emigrate at any time and thus without synchrony*	[31]
	c	successful immigrants should settle before the start of breeding period, that is no later than April	[32,33,52]
P3	**a**	**juveniles should travel for longer periods of time than adults during the transience stage**	[2,16–18,22–25,53]
	b	*juveniles should travel over greater distances than adults during the transience stage*	
	c	there should not be any difference between age groups regarding the linearity of movements during the transience stage	
	d	*there should not be any difference between age groups regarding the daily rate of movements during the transience stage*	
**P4**		**sex should not affect the occurrence of emigration and successful immigration, the timing of emigration and immigration, or the various movement metrics measured during transience**	[2,52,54,55]

## 2. Material and methods

### 2.1. Study site

We worked in the south plain of Bylot Island (73°N, 80°W), which is part of Sirmilik National Park, Nunavut, Canada. Between 100 and 110 fox dens were monitored annually in our 600 km^2^ study area [56]. At this site, the Arctic fox is the main tundra predator [57] and its prey base consists mostly of brown (*Lemmus sibiricus*) and collared lemmings (*Dicrostonyx groenlandicus*), together with the greater snow goose (*Chen caerulescens atlantica*) [58]. Bylot Island is surrounded by sea ice from late October to late July [59]. It is part of the Canadian Arctic Archipelago which comprises around 36 500 islands separated by many waterways, straits and channels spanning over 1900 km from north to south and 2400 km from east to west [60].

### 2.2. Capture and tracking of individuals

We obtained locations of Arctic foxes between May 2007 and September 2021 by tracking individuals carrying collars equipped with Argos Platform Terminal Transmitters (models KiwiSat 202 and 303, Lotek, Newmarket, Ontario, Canada; 95 g–115 g). We collared 148 adults (72 females and 76 males) and 22 juveniles (8 females and 14 males) during 13 summer field seasons in 2007–2019, using capture methods described in [42]. Argos collars transmitted daily from 13:00 or 14:00 to 17:00 UTC with a repetition rate of 60 s. Transmission schedule was different for 10 collars that transmitted every second day from 16 August–31 May (2007–2008) and for 12 collars that transmitted every second day from 16 May–14 October (2009–2011). We only used the most accurate Argos positions, those with location classes LC3, LC2 and LC1, which respectively correspond to positioning errors having a 68% probability of being <250 m, <500 m and <1500 m [61]. Retained locations were then passed through a speed filter implemented in R 3.6.3 [62,63] so that any location requiring unrealistic speed values from the previous one was removed. Speed values (7 km h^−1^ cruising speed, with possible 12 min acceleration bouts of 10 km h^−1^ for locations less than 10 min apart) were based on data obtained from GPS collars in the same fox population [62]. One location per tracking day was kept, based on the smallest location error. We mapped locations using QGIS 3.10 [64].

### 2.3. Characterization of long-distance dispersal events

We first verified that individuals were resident (lived in a territory) at the start of their tracking. For juveniles, we simply ensured that captures occurred in the natal territory. For adults, we applied a 5 km radius buffer around the mean of the first two weeks of locations after capture and only retained for analysis individuals with greater than or equal to 50% of these locations within this buffer (hereafter called ‘territorial buffer’). Other individuals were considered transients and removed from analyses. Buffer size was slightly larger than the average radius of an Arctic fox home range on Bylot Island (approx. 3.5 km) [65] to accommodate short extraterritorial excursions.

#### 2.3.1. Emigration

Arctic foxes can perform extraterritorial movements as part of their foraging activities [40], but these movements include a return to the territory and are thus easily separated from dispersal. In a detailed analysis of movements in the study population [40], it was observed that extraterritorial excursions mainly remained within 80 km from the territory. In the larger red fox (*Vulpes vulpes*) studied in Scandinavia, individuals dispersing straight-line distances >60 km represented outliers on the spectrum of dispersal distances [66]. We, therefore, considered any extraterritorial movement that reached a distance of greater than or equal to 80 km away from the boundary of the buffer during the movement event and with no permanent return to the territory as long-distance dispersal. We identified emigration date as the date of the last location in the initial territorial buffer.

#### 2.3.2. Immigration

The establishment of a new territory following long-distance dispersal was identified by a final sedentary stage, corresponding to greater than or equal to 50% of locations of an individual remaining in a 5 km radius buffer for greater than or equal to 1 month. The proportion of locations and the size of the radius buffer mirror the criteria used to confirm that individuals were sedentary at capture, and using a one-month rather than two-week period to confirm the final sedentary phase allowed us to exclude stop-overs sometimes observed during transience. We identified immigration date as the date of the first location in the final territorial buffer. The long-distance dispersal was considered complete when the fox established a new territory and incomplete when the tracking stopped during transience because the fox died or the collar failed.

Calculated emigration and immigration dates were confirmed by visualizing dispersal movements using QGIS v. 3.10 [64].

### 2.4. Calculation of long-distance dispersal metrics and statistical analyses

Once emigration and immigration dates (or dates when tracking was interrupted by death or collar failure) were obtained, transience of each individual was quantified by calculating its duration (number of days), straight-line distance (Euclidian distance between initial territorial buffer borders and last location of transience), minimum distance travelled (sum of straight-line distances between successive locations obtained from initial territorial buffer and last location of transience), linearity (straight-line distance divided by minimum distance travelled), initial bearing (angle between first location of transience and the first location greater than or equal to 50 km from initial territorial buffer borders), final bearing (angle between first and last locations of transience) and minimum average daily movement rate (minimum distance travelled divided by number of travelling days).

We used Chi-squared tests to compare proportions of occurrence of long-distance dispersal and immigration between age and sex groups (P1a-b; P4). To incorporate the circular nature of the timing data (December being close to January), we used Watson's U^2^ tests (package circular v. 0.4–93 in R) to compare the circular distributions of the timing of emigration and immigration (P2a–c). The package circular returns for the Watson's test a range of *p*-values, rather than exact *p*-values. We compared movement metrics across age and sex groups using Student's or Welch's *t*-tests (package stats v. 3.6.2 in R), depending on the equality of variances, and performed a permutation *t*-test when condition of normality was not met (P3a–d; P4). We used Rayleigh tests (package circular v. 0.4–93 in R) to determine the presence of significant directional angles at the beginning (initial bearing) and at the end (final bearing) of long-distance dispersal.

### 2.5. The challenges of studying long-distance dispersal

A full appreciation of our results requires a good understanding of how we minimized three fundamental problems regarding the study of long-distance dispersal, namely its weak definition, the narrative and qualitative nature of most of its reports, and the sensitivity of its measures to the scale of methods [7].

First, we used an absolute distance definition of long-distance dispersal, specifically dispersal over an 80 km threshold. Absolute distance definitions refer to an ecologically relevant scale, for example, the distance over which adults interact and reproduce [67–69]. Our choice of threshold was based on *a priori* knowledge of the spatial ecology of the studied population [40] and seemed conservative for a 3 kg terrestrial mammal living in small territories, especially given the 60 km threshold that was found to be adequate for red foxes [66]. An 80 km threshold is also in good line with the spatial scale of long-distance dispersal in many other organisms (table 1 in [7]), with the notable exception of bird.

Second, satellite tracking is now a widely used method to track wildlife dispersers, particularly carnivores [17,66,70]. Our large investment in satellite tracking (170 individuals receiving 191 collars) made it possible to quantify many metrics in the studied population, thus moving away from earlier natural history reports often limited to describing extensive movements in this species [38,39,41]. Our large sample sizes also allowed us to compare some long-distance dispersal metrics across age and sex classes, although not without statistical limitations.

Third, while the relevance of long-distance dispersal studies can be limited by their spatial and temporal scales, the global coverage of the Argos satellite constellation [71] allowed us to track foxes irrespective of their geographical location, thus removing spatial biases in disperser detection. Furthermore, interannual variability in frequency and spatial extent of long-distance dispersal was smoothed by the length of our 13-year study, while intra-annual variability in individual movements was captured by the long (14 months) expected battery life of satellite collars. This minimized temporal biases in disperser detection, and our results should be rather robust to problems caused by sampling designs with inadequate spatio-temporal scales.

## 3. Results

### 3.1. Occurrence and immigration success of long-distance dispersal

**Figure 1.**
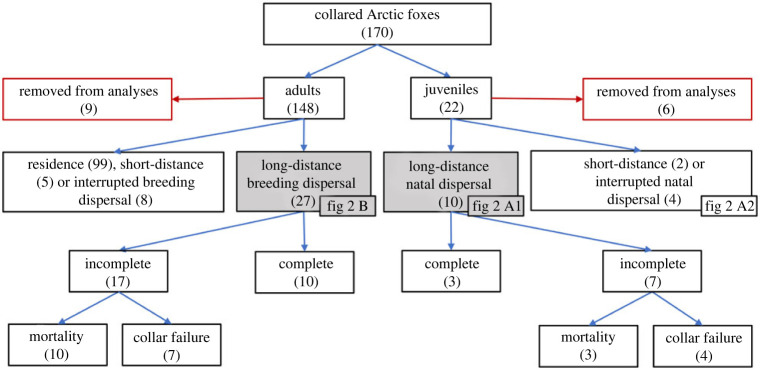
Fate of 170 Arctic foxes equipped with Argos Platform Terminal Transmitters on Bylot Island (Nunavut, Canada) in 2007–2019 and tracked from 2007–2021. Long-distance dispersal was defined as a movement with no permanent return reaching ≥80 km away from the boundary of the initial territorial buffer where they resided when captured. Unique ID and individual fate of all individuals are reported in the electronic supplementary material, table S2.

**Figure 2.**
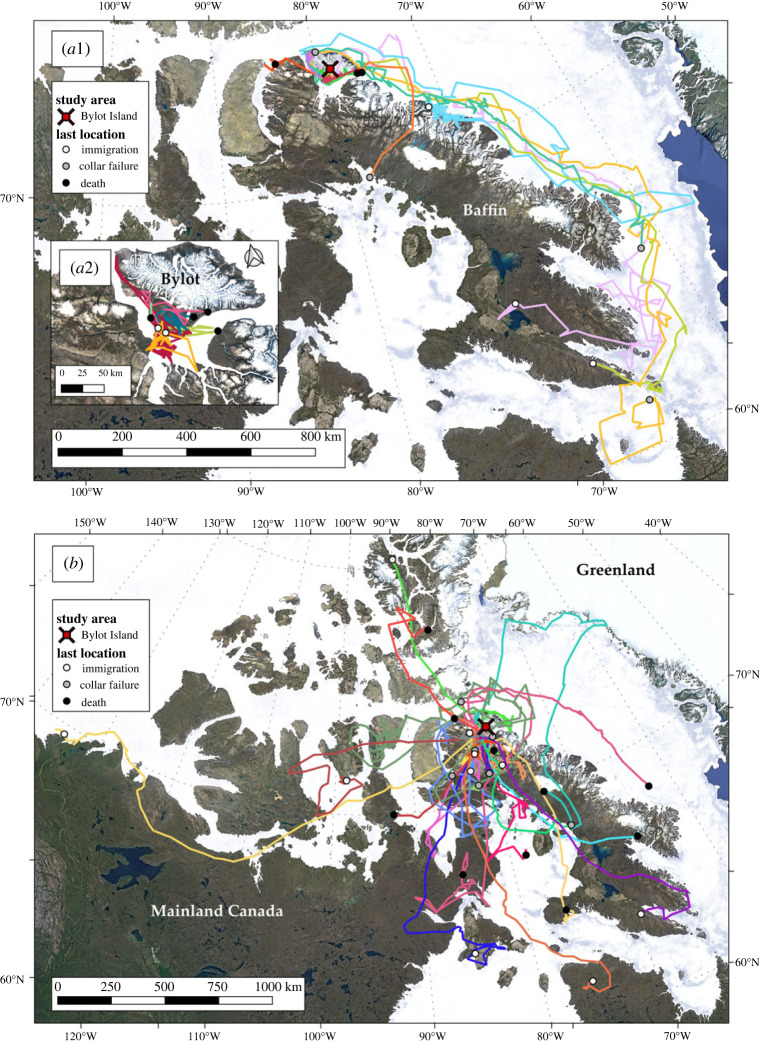
Long-distance dispersal paths of juveniles (*a*1; *n* = 10) and adults (*b*; *n* = 27) from Bylot Island between 2007 and 2021. Paths of interrupted natal dispersal (*n* = 4) and short-distance natal dispersal (*n* = 2) are presented in the inset map (*a*2). Dots at the end of the paths indicate successful dispersal with immigration (white) or tracking ending during transience by collar failure (gray) or fox death (black). *a*1 mostly shows Baffin Island, *a*2 mostly shows Bylot Island (located at the northern tip of Baffin), and B mostly shows the Canadian Arctic Archipelago, located between mainland Canada and Greenland.

Of the 170 collared Arctic foxes, 4 (2 adults, 2 juveniles) had their collar failing within two weeks of capture, 7 adults were transients when captured and 4 juveniles died in their natal range during the first two months of tracking, thus leaving 155 individuals for further analyses (figure 1, top two rows). Of these, 10 juveniles (1 female, 9 males) and 27 adults (12 females, 15 males) undertook long-distance dispersal (figure 1, third row, and figure 2, *a*1 and *b*). The transience stage of all long-distance dispersal can be visualized in electronic supplementary material, video S1, a dynamic map available in electronic supplementary material. A larger proportion of juveniles (62.5%) than adults (19.4%) engaged in long-distance dispersal ($\chi_{2}^{2}=44.36$ , *p* < 0.001), thus supporting P1a. In addition, interrupted natal dispersal events were suggested for 4 juveniles (2 females, 2 males) that died shortly after leaving the den site but <80 km away from their natal range (figure 2*a*2). The two remaining juveniles (1 female, 1 male) established a new home range near their natal territory (short-distance dispersal) (figure 1, third row, figure 2*a*2). Finally, short-distance breeding dispersal events were suggested for 5 adults (5 females), interrupted breeding dispersal events for 8 adults (5 females, 3 males) and the remaining individuals (45 females, 54 males) were considered as residents (figure 1, third row). Note that interrupted breeding dispersal events could also be interrupted extraterritorial excursions, but the death of the individual (2 females, 1 male) or the failure of the collar (3 females, 2 males) made it impossible to separate the two options.

Among the foxes undertaking long-distance dispersal, only 3 juveniles (3 males) and 10 adults (6 females, 4 males) settled in a new territory (figure 1, fourth row). The proportion of juveniles (30%) managing to settle after performing long-distance dispersal did not differ from that of adults (37%) ($\chi_{1}^{2}<0.001$; *p* = 0.992), thus contradicting P1b. Note that among individuals engaging in long-distance dispersal but for which settlement was not recorded, the proportion of cases ending with a documented death was relatively similar for adults (58%) and juveniles (43%) (figure 1, bottom row).

### 3.2. Timing of emigration and immigration

**Figure 3.**
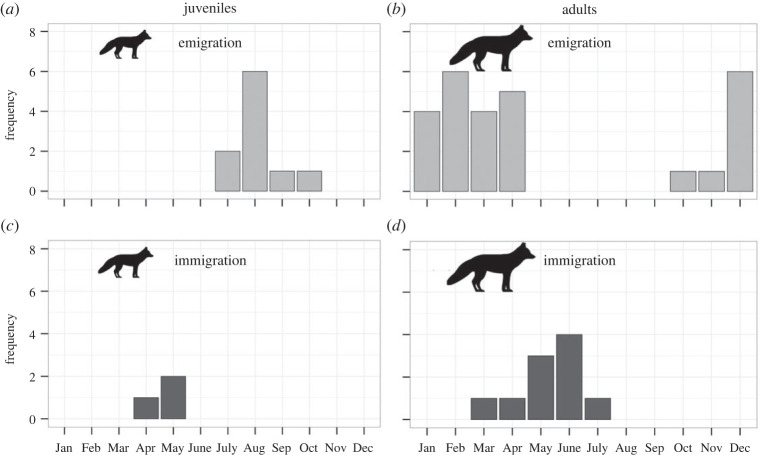
Timing of emigration (light gray) in juvenile (*a*; *n* = 10) and adult (*b*; *n* = 27) Arctic foxes and timing of immigration (dark gray) in juvenile (*c*; *n* = 3) and adult (*d*; *n* = 10) Arctic foxes undertaking long-distance dispersal after being equipped with Argos Platform Terminal Transmitters on Bylot Island (Nunavut, Canada) in 2007–2019.

Emigration dates of juvenile long-distance dispersal were spread from July to October and clearly peaked in August, with no event taking place from November to June (figure 3*a*), thus strongly supporting P2a. Emigration dates of adult long-distance dispersal occurred from October to April without peaking at any particular time, and no event took place from May to September (figure 3*b*), which only partially supports P2b. Distributions of emigration dates clearly differed across age groups (Watson's *U*^2^ = 0.606, *p* < 0.001).

Immigration dates were strongly concentrated in spring (figure 3*c*,*d*). Immigration of juveniles following long-distance dispersal occurred in April–May (figure 3*c*) but sample size was too small (*n* = 3) to allow robust inference regarding P2c. Immigration of adults following long-distance dispersal peaked in May–June (figure 3*d*), too late to support P2c. No immigration occurred between August and February. The distributions of immigration dates through time were similar across age groups, although larger sample sizes might have revealed differences (Watson's *U*^2^ = 0.159, 0.05 < *p* < 0.10).

### 3.3. Movement metrics during transience

Juveniles performing long-distance dispersal mostly travelled along the eastern coast of Baffin Island (figure 2*a*1), while none of the adults performing long-distance dispersal followed such a trajectory (figure 2*b*). Adults rather travelled in all directions, some of them reaching Ellesmere Island, Greenland, Quebec and the Yukon border. The transience stage lasted significantly longer in juveniles than adults (table 2 for all *p* values), hence supporting P3a (table 2). This result held true whether only complete or all long-distance dispersal were considered, and effect sizes were large (ca. 3 months versus ca. 5.5 months on average in adults/all long-distance dispersal versus juveniles/all long-distance dispersal, table 2). There was strong evidence for the minimum distance travelled during transience to be larger in juveniles than adults when complete long-distance dispersal were considered (on average 4804 km versus 1853 km, table 2), thus supporting P3b. However, when all long-distance dispersal were considered, or when straight-line distances were compared, there was no evidence for travelled distances between juveniles and adults to differ, thus weakening support to P3b. Juveniles clearly travelled in a less linear way than adults during transience (table 2), contradicting P3c. Regarding average movement rate (P3d), the evidence was unclear, with adults travelling almost twice as fast as juveniles when all long-distance dispersal were considered, but at a similar rate when only complete long-distance dispersal was considered (table 2).

**Table 2.** Mean (± s.e.) and range [Min - Max] of movement metrics during the transience stage of long-distance dispersal for juvenile and adult Arctic foxes from Bylot Island (Nunavut, Canada). Tested predictions appear on the left column. Data from long-distance dispersal events followed by successful settlement (Complete) are shown separately to allow more meaningful data interpretation. ‘All’ includes complete tracks and tracks interrupted by collar failure or fox death. Results of statistical analyses (two right columns) compare metrics of natal and breeding dispersal. Superscripts indicate statistical significance (*), use of a permutation test because of non-normality of the data (^1^), or sample size reduced to *n* = 26 for adults/All (^2^).

		juveniles		adults		juveniles versus adults	
		complete (*n* = 3)	all (*n* = 10)	complete (*n* = 10)	all (*n* = 27)	complete (*n* = 13)	all (*n* = 37)
P3a	duration (days)	247.0 *(± 27.4*)	167.8 (*± 25.0*)	87.6 (*± 15.3*)	89.8 (*± 16.4)*	*t_11_* = −5.02	*t_35_* = −2.90
		[206–299]	[45–299]	[15–202]	[12–376]	*p* < 0.001 *	*p* = 0.006 *
P3b	straight-line distance (km)	822.3 (*± 266.7*)	582.7 (*± 169.3*)	746.0 (*± 194.1*)	545.5 (*± 88.4*)	*t_11_* = −0.20	*t_35_* = 0.21
		[321.8–1232.3]	[83.8–1446.2]	[66.3–1898.1]	[66.3–1898.1]	*p* = 0.848	*p* = 0.840
	minimum distance travelled (km)	4804.0 (*± 943.2*)	2470.3 (*± 678.5*)	1853.5 (*± 246.7*)	1604.1 (*± 241.7*)	*t_11_* = −4.52	*t_35_* = −0.86
		[3160.7–6428.0]	[398.6–6428.0]	[221.5–2968.6]	[186.6–6137.3]	*p* < 0.001 *	*p* = 0.394
P3c	linearity	0.20 (*± 0.10*)	0.25 (*± 0.04*)	0.38 (*± 0.08*)	0.40 (*± 0.05*)	*t_11_* = 1.23	*t_32_._2_* = 2.56 ^1^
		[0.07–0.39]	[0.07–0.39]	[0.06–0.68]	[0.03–0.88]	*p* = 0.066	*p* = 0.015 *
P3d	average daily movement rate (km day^−1^)	19.7 (*± 3.4*)	13.0 (*± 2.2*)	23.7 (*± 2.7*)	23.4 (*± 2.0*)	*t_11_* = 0.73	*t_35_* = 2.98
		[13.0–23.9]	[4.6–23.9]	[6.7–36.6]	[5.4–37.7]	*p* = 0.478	*p* = 0.005 *
	*On land* ^2^	15.5 (*± 4.4*)	9.7 (*± 1.8)*	28.2 (*± 3.8*)	27.5 (*± 3.2*)	*t_11_* = 1.72	*t_23_._9_* = 4.18 ^1^
		[6.9–21.3]	[4.4–21.3]	[6.0–44.4]	[2.7–80.6]	*p* = 0.114	*p* < 0.001 *
	*On sea ice* ^2^	22.8 (*± 2.1*)	17.7 (*± 2.1*)	20.0 (*± 3.5*)	21.7 (*± 2.1*)	*t_11_* = −0.43	*t_26_._3_* = 1.39 ^1^
		[19.3–26.5]	[4.8–26.5]	[5.4–37.3]	[5.4–37.3]	*p* = 0.678	*p* = 0.177
	average percentage of locations on sea ice (%)	60.3 (*± 5.5*)	40.1 (*± 8.3*)	38. 9 (*± 5.6*)	43.5 (*± 4.8*)	*t_11_* = −1.97	*t_35_* = 0.37
		[49.8–68.0]	[3.1–73.0]	[5.1–62.9]	[0.0–100.0]	*p* = 0.075	*p* = 0.718

### 3.4. Sex effects

In adults, there was no evidence that sex influenced the occurrence of long-distance dispersal (females (*n* = 12): 17.9% versus males (*n* = 15): 20.8%, $\chi_{1}^{2}=0.049$, *p* = 0.825) or the proportion of complete long-distance dispersal (females (*n* = 6): 50.0% versus males (*n* = 4): 26.7%, $\chi_{1}^{2}=0.72$, *p* = 0.397), thus supporting P4. The metrics shown in table 2 were also tested for sex effects, with no significant differences found (electronic supplementary material, table S1), again supporting P4. Sex biases (P4) could not be tested for juveniles given that only one juvenile female undertook long-distance dispersal.

### 3.5. Direction of travel and use of sea ice during long-distance dispersal

**Figure 4.**
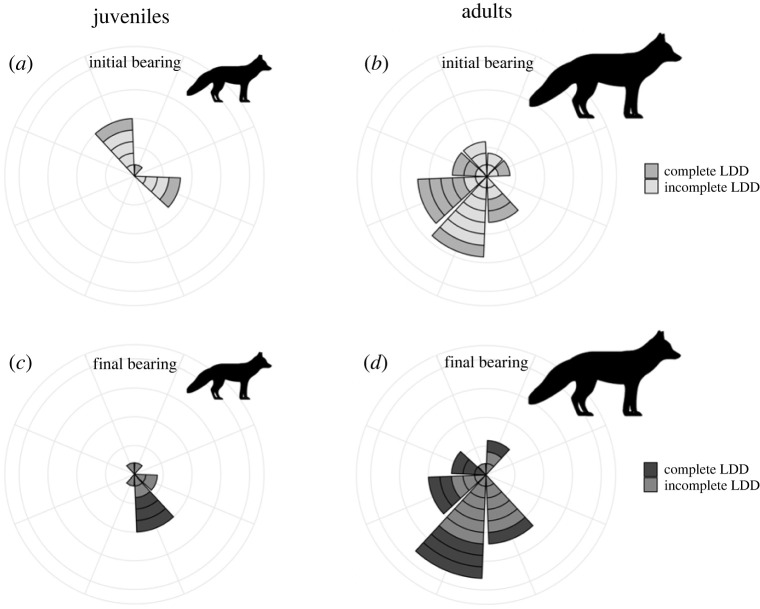
Initial and final bearings for complete and incomplete long-distance dispersal (LDD) undertaken by juvenile (initial: *a*, final: *c*, *n* = 10) and adult (initial: *b*, final: *d*, *n* = 27) Arctic foxes tracked from Bylot Island (Nunavut, Canada) in 2007–2019.

The analysis of travel directions of individuals performing long-distance dispersal indicated that bearings were generally not uniform (table 3). When considering all long-distance dispersal, upon departure, juveniles mainly took initial bearings to the northwest or east (figure 4*a*), but most settled or ended their course at a final bearing to the southeast (figure 4*c*). On the contrary, most departing adults initially headed southward (figure 4*b*) and approximately kept that heading until the end of their course (figure 4*d*). Results showed a similar trend when only complete long-distance dispersal was analysed, although not statistically significant, perhaps due to much reduced sample sizes (table 3).

**Table 3.** Mean angle in degrees (*± SD*) and mean resultant length and its *p* value obtained from the Rayleigh test (mean resultant length varies between 0 and 1, where 1 means few variations between the bearings) when analysing the initial and final bearings of juvenile and adult Arctic foxes undertaking long-distance dispersal from Bylot Island (Nunavut, Canada). Data from long-distance dispersal events followed by successful settlement (Complete) are shown separately to allow more meaningful data interpretation. ‘All’ includes both complete tracks and incomplete tracks interrupted by collar failure or fox death. Superscripts (*) highlight when angle distribution is significantly different from randomness.

	juveniles		adults	
	complete (*n* = 3)	all (*n* = 10)	complete (*n* = 10)	all (*n* = 27)
initial bearing	8.3° *(± 0.7*)	14.8° (*± 0.9*)	−157.4° (*± 0.8*)	150.2° (*± 1.0*)
	0.62 (*p* = *0.354*)	0.56 (*p* = *0.040*) *	0.56 (*p* = *0.039*) *	0.41 (*p* = *0.009*) *
final bearing	138.8° *(± 0.2*)	124.3° (*± 0.9*)	−139.3° (*± 0.9*)	−163.7° (*± 0.9*)
	0.95 (*p* = *0.052*)	0.55 (*p* = *0.042*) *	0.52 (*p* = *0.063*)	0.05 (*p* = *0.001*) *

Both juveniles and adults generally spent a considerable amount of time on the sea ice during long-distance dispersal, but there was no evidence that average percentage of time spent on land versus sea ice differed across age groups (table 2). When considering all long-distance dispersal, juveniles travelled at a higher rate on sea ice than on land (*t* = 2.89, *p* = 0.010), but that was not the case when considering complete long-distance dispersal (*t* = 1.52, *p* = 0.204). Adults travelled at the same rate whether on land or sea ice (All long-distance dispersal, *t* = −0.83, *p* = 0.412; Complete long-distance dispersal, *t* = −1.60, *p* = 0.127). When daily movements rates on the same medium (land or sea ice) for both age classes were compared, adults moved two to three times faster on land than juveniles, a trend that was statistically significant when all long-distance dispersal were considered (table 2). However, both adults and juveniles travelled at the same daily movement rate when on the sea ice (table 2).

## 4. Discussion

Our long-term satellite tracking of juvenile and adult Arctic foxes from Bylot Island allowed us to monitor multiple long-distance dispersal events and test detailed hypotheses about an animal behaviour that is remarkably difficult to study. We first evaluate the relevance of our findings regarding Arctic fox ecology, then discuss how this study advances our understanding of long-distance dispersal in animals.

### 4.1. Significance of long-distance dispersal for Arctic fox ecology

Using an extended dataset, we confirmed previous findings (figure 2 in [40], where long-distance dispersal was called ‘nomadism’) that a high proportion of dispersing adults in our study population disperse over long distances. Whereas the prevalence of long-distance dispersal is difficult to compare across species because of the various ways in which it is defined, this behaviour seems exceptionally high in Arctic foxes. Indeed, several published records of long movements by terrestrial animals come from this species studied in Alaska [41,72], Canada [39] and Svalbard [38].

Phylogeny cannot explain the high prevalence of long-distance dispersal in Arctic foxes because the two most closely related species, the kit fox (*V. macrotis*) and the swift fox (*V. velox*) [73,74] do not show high rates of this type of movement [75,76]. Ecological conditions are a more likely driver since other populations show low rates of long-distance dispersal, such as in Iceland [77] where food resources are abundant and reliable, and unoccupied dens are abundant [54]. Hypotheses for the high long-distance dispersal rate from Bylot could involve high local fox densities, food resources that are highly heterogeneous in space and time, and a virtual absence of barriers to fox movements during most of the year [78–81].

As predicted (P1a), the occurrence of long-distance dispersal was far greater in juveniles than in adults, following the trend observed in most animal species. Breeding dispersal is positively correlated with age in our study population [40], the oldest individuals potentially being the least able to defend their territory against competitors. It is surprising that immigration success was similar for juveniles than adults (P1b contradicted), since the broader experience of adults should facilitate their establishment, as observed in swift foxes [29]. However, as adults dispersing from Bylot tend to be the oldest, and thus potentially less competitive individuals [40], senescence may remove the advantages that experienced adults have over juveniles. Immigration success was generally low for juveniles and adults performing long-distance dispersal. This is coherent with the costs associated with long-distance dispersal, but the interpretation of our results is limited by our inability to evaluate reproductive success after settlement [82].

As predicted (P2a), emigration was seasonal and well synchronized in juveniles, but not in adults. The July–October pattern of emigration observed in juveniles fits data from red and kit foxes [75,83], where departures often correspond to an increase in littermate aggressions as juveniles become independent in late summer. Bimodal patterns of emigration were observed in other canids, where a second peak was triggered by aggressions from adult competitors during the breeding season [28,29,84,85]. We did not observe this second peak, perhaps due to reduced sample sizes. The pattern observed in adults, with emigration dates spread from October–April (P2b partially supported), suggests that emigration is relaxed during the breeding season when adults invest in parental care [86,87] and resource availability is at its highest [58]. The degraded conditions of travel on sea ice in July–October may also help explain adult emigration phenology, thus fitting the hypothesis that emigration is postponed until conditions optimize dispersal success [88,89]. Note that juveniles did emigrate before ice formed around Bylot in November; their initial phase of transience was thus limited to the island, resulting in restricted movement patterns.

Immigration phenology was similar between age classes and concentrated in May–June (9 of 13 observations). This was too late to allow birthing and pup rearing in a den, thus contradicting P2c. This observation could reflect the difficulty to find a new territory for most foxes performing long-distance dispersal, highlighting its high cost and again suggesting that it could result from the intense competition in the source population.

The differences in long-distance dispersal metrics between juveniles and adults during transience are of high interest given the paucity of literature on this matter. Minimum distances travelled were extremely high, reaching thousands of km in both juveniles and adults. The largest minimum distances travelled were >6000 km in both age classes, well over previous records (e.g. 3506 km [38]). The longer duration of transience observed in juveniles than adults (P3a) fits their earlier emigration but rather similar immigration dates. Such a longer duration of transience translated into a larger overall distance travelled by juveniles (P3b), but not into a larger distance between their source and destination territories. This is to be directly related to their initial phase of transience restricted to Bylot Island because of the absence of sea ice in fall. This restricted movement pattern may also explain that juveniles overall travelled in a less linear way and tended to show smaller average daily movement rates than adults (P3c and P3d contradicted). We therefore suggest that the observed differences in transience metrics between juveniles and adults are at least partially due to the insular situation and temporal dynamics of sea ice. Several other Arctic fox populations are coastal and depend on sea ice during parts of their life cycle [90], so our results could inspire new hypotheses regarding dispersal patterns in other populations.

We found no sex difference in long-distance dispersal metrics, as predicted (P4). Even if dispersal is generally male-biased in mammals, males and females can disperse evenly in monogamous species [2,55]. This is also supported by earlier works on wolves [91] and some Arctic fox populations from Iceland and Sweden [77]. However, in some Arctic fox populations, juvenile females disperse less often and over shorter distances than males [92,93], reflecting the high flexibility of Arctic fox social organization and dispersal [92,94]. Patterns may therefore differ between populations, depending on local ecological conditions. Larger sample sizes, particularly for juveniles, will be needed to robustly address this question.

The strong gene flows detected between Arctic fox subpopulations linked by sea ice [43,44,95] has long shown that individuals disperse across sea ice. Our finding that foxes engaging in long-distance dispersal from Bylot Island spent nearly half of their transience time on sea ice is thus not surprising. It confirms that decreasing sea ice extent and duration will affect Arctic fox movements and genetic structure [96]. A more surprising finding, however, is that juveniles and adults used different dispersal routes, juveniles being much more likely than adults to travel on the sea ice adjacent to the east coast of Baffin Island. When combined with the different long-distance dispersal phenology of juveniles and adults, this suggests that natal and breeding dispersal might be differentially affected by climate warming as connectivity between Arctic land masses is disturbed [97].

The Arctic fox is the main reservoir and vector of the Arctic rabies virus variant [98], a zoonotic disease potentially fatal to humans. Little is known, however, about the ecological mechanisms driving where and when rabies occur in fox populations [98,99]. Our results shed light on this problem in four ways. First, the high proportion of individuals undertaking long-distance dispersal explains that Arctic foxes can spread the rabies virus efficiently across the Arctic. Second, the scale of observed movements confirms that this species can spread the virus at a continental scale, as suggested by temporal correlations in the number of rabies cases between adjacent areas across northern Canada [100]. Third, whereas juvenile dispersal is traditionally seen as a main building block for the virus spread, we show that the role of adults should not be underestimated, especially in efforts to model rabies outbreaks [101]. Finally, genetic analyses suggest that rabies spreads among fox populations via the dispersal of rabid individuals from north to south [102]. This hypothesis is supported by the general southward orientation of long-distance dispersal observed from Bylot Island. The higher tundra productivity in the southern than in the northern Arctic, and the geographical barriers (notably Lancaster Sound) north of Bylot could explain this pattern. To close, although much less is known about the epizootiology of *Echinococcus multilocularis* than that of the rabies virus, it will be useful to investigate how our findings help to understand the transmission of this tapeworm which causes fatal liver infection in humans [103].

### 4.2. Determinants and outcomes of long-distance dispersal in animals

This Arctic fox case study contributes to our understanding of long-distance dispersal in animals as it reduces the gap between observations and theory. It notably helps to better understand the transience stage since we could monitor complete paths despite the large efforts required for tracking highly mobile species. Our empirical data allow us to highlight differences from theoretical models, which often assume that individuals distance themselves from their starting point at a constant rate and in a constant direction [104]. The presence of temporary stop-overs, quick returns near the initial area and other direction changes bring interindividual variability that should be considered in the study of transience patterns [66].

Although the benefits of undertaking long-distance dispersal might be predictable based on environmental characteristics, not all individuals can afford the costs of long-distance movements, thus explaining the persistence of different dispersal strategies [105,106] in our system. We confirmed that this movement can be influenced by life-history traits such as age class. However, it would be interesting to investigate how other individual characteristics, like body condition and personality, could influence the probability and metrics of long-distance dispersal [1,14,107].

Long-distance dispersal may represent a high-cost high-gain strategy for individuals, but there could also be population- and community-level implications [15], such as the rate of spread of an expanding population [108,109], the connectivity among populations [110], and the spatio-temporal patterns of genetic diversity [111]. Regarding the latter, unlike short-distance dispersal where the pure diffusion spread should lead to an increase in genetic differentiation along the expansion range [112], long-distance dispersal has the potential to attenuate genetic differentiation by proceeding in allele exchanges between core and peripheral populations [113]. Although this is modulated by the relative frequency of long-distance dispersal, this has the potential to lead to panmictic populations where genetic variability is low. Our results confirm the important connectivity that can result from this large-scale movement [43–45].

## Data Availability

All Arctic fox Argos locations are available through the Movebank Data Repository (Movebank Study ID 942774711) at https://www.movebank.org/cms/webapp?gwt_fragment=page=studies,path=study942774711. Data and relevant code for this research work are stored in GitHub: https://github.com/chaireBioNorth/ArcticFoxLDD and have been archived within the Zenodo repository: https://doi.org/10.5281/zenodo.7521679 [114]. The data are provided in electronic supplementary material [115].
